# Effects of aquatic and land high-intensity interval trainings on selected bio- and physiological variables among obese adolescents

**DOI:** 10.3389/fendo.2024.1381925

**Published:** 2024-09-27

**Authors:** Ting Liao, Chuanbo Zheng, Jungang Xue, Yong “Tai” Wang

**Affiliations:** ^1^ Aquatic Therapy and Fitness Research Centre, Wuhan Sports University, Wuhan, China; ^2^ College of Health Sciences and Technology, Rochester Institute of Technology, Rochester, NY, United States

**Keywords:** high-intensity interval trainings, aquatic high-intensity interval trainings, overweight, obese, adolescents

## Abstract

**Background:**

Obesity among adolescents have become a global public health problem. Exercises can effectively improve the bio-physiological factors of obese adolescents. High-intensive interval training (HIIT) has been applied to obese adolescents. Studies have reported that the Aquatic environment may bring the same or more positive exercise effects as the land environment. Therefore, the purpose of this study was to examine the effects of aquatic and land interventions on selected bio-and physiological variables among obese adolescences.

**Methods:**

Twenty-eight obese adolescents who met the requirements participated in and completed this study. The participants were randomly assigned to Aquatic HIIT group (n=17) or Land HIIT group (n=11) for a four-week exercise intervention, 3 time/week. Each Intervention program was one-hour long, including 20 minutes of warm-up, 30 minutes of HIIT and 10 minutes of stretching and relaxation. Bio- and physiological variables including Anthropometry and body composition, Physical Function and blood pressure, and Lipid metabolism indexes were collected before and after the Aquatic and Land interventions.

**Results:**

After four weeks of exercise interventions, the body mass, BMI, body fat rate, waist circumference, hip circumference and body water content were significantly reduced (*p*<0.05), and the lean body mass were significantly increased (*p*<0.05) in both groups. Both group exhibited significant effects in decreasing, systolic blood pressure (*p*<0.05), diastolic blood pressure (*p*<0.01), and increasing vital capacity and total energy consumption (*p*<0.05). The Aquatic HIIT group showed significant effects on reducing Rest heart rate (*p*<0.05), but no significant changes in Rest heart rate in Land HIIT group (*p*=0.364). The low-density lipoprotein cholesterol in both groups was significantly decreased (*p*<0.05). Moreover, the Aquatic HIIT group had significant better improvements (*p*<0.05) in lean body mass, waist circumference, waist-to-hip ratio, vital capacity and total energy consumption than Land HIIT group did.

**Conclusions:**

The results of the present study demonstrated that in a short-term (4 weeks) both Aquatic and Land HIIT interventions may improve the body composition, physical function, blood pressure and low-density lipoprotein cholesterol (LDL-C) of overweight and obese adolescents. Furthermore, the Aquatic HIIT may be superior than the Land HIIT in weight control among the obese adolescents.

## 1 Introduction

Nowadays, adolescent obesity has become a global public health challenge in the 21st century, and overweight and obesity are caused by multi-factors which include behaviors like eating patterns and lack of adequate physical activity or sleep, and genetics and family history (1). Obese adolescents have a higher incidence of hypertension, non-alcoholic fatty liver disease (NAFLD), obstructive sleep apnea (OSA), dyslipidemia, cancer, diabetes, metabolic syndrome, and cardiovascular disease compared to their normal peers (2, 3). Psychologically, obese adolescents are at higher risk for major depression (4), anxiety and mood regulation disorders (5). Studies have shown a significant correlation between adolescent obesity rates and lack of physical activity (6). Moderate to vigorous physical activity can lead to a variety of positive health outcomes (7), and can effectively prevent or offset that development of obesity (8). Therefore, exercise interventions have been increasingly used to improve adolescent obesity.

In the past, high intensity interval training (HIIT) was known for the characteristics of repeatedly performing short-time high-intensity activities, which required performing physical activities with close to maximum or full effort, corresponding to exercise intensity ≥90% of maximal oxygen uptake or > 75% of maximal power, and performing short-time passive rest or active recovery between each group of exercises (9, 10). Previous studies have shown that the application of HIIT in weight management can reduce the blood pressure (11), weight, body mass index (BMI) and body fat rate of adults (12), as well as the lean body weight (13), waist circumference (14), hip circumference (14) and waist-hip ratio of adolescents (15). Moreover, HIIT showed significant effect in improving triglycerides (TG), total cholesterol (TC), low-density lipoprotein cholesterol (LDL-C) and high-density lipoprotein cholesterol (HDL-C) of the obese and overweight populations (16). In addition, the HIIT also has a positive effect on improving cardiorespiratory function (17) and maintaining cardiovascular health (18) in adolescents. Recently, the HIIT has been considered as an effective alternative to traditional continuous training among exercise interventions for weight loss (19, 20). Previous studies have found that a long-term HIIT (8-12 weeks) can reduce LDL-C (21), body fat reduction (22), and body mass (23) in obese people, and a short-term HIIT (4 weeks) has also shown a positive promise that the body mass could be reduced (24).

People who are overweight or obese during land exercise generally may face a high risk of bone and joint injury, limb mobility restrictions and falls (25, 26). HIIT programs such as high-intensity “uphill”, treadmill running or brisk walking are more likely to cause secondary injuries, such as meniscal injury (27) and plantar fasciitis (28) due to the repetitive impact movements. Land exercise may also cause physical discomforts for obese individuals, include excessive joint pressure (29) and thermal discomfort (30). Yet, the non-weight and low-impact HIIT may be more practical for improving the condition of overweight and/or obesity (31).

Due to the unique characteristics of the aquatic environment, aquatic exercise can prevent muscle damage (32, 33), reduce body weight load, relieve swelling, joint wear and pain, and increase body flexibility and mobility (34–36). In recent years, aquatic exercise intervention has been implemented on obese adults (37) and adolescents (38) on weight loss, and its effectiveness in improving the body composition of obese people has been confirmed (39, 40). The attrition for the aquatic exercise or intervention is lower than that of the land exercise or intervention (38), which indicated that aquatic exercise intervention may have significant potential and value in alleviating exercise discomfort of obese people and reducing the risk of exercise injury. Compared with land HIIT, aquatic HIIT may have advantages in terms of improving respiratory pressure and blood vessel pressure (41), and achieve higher intensity along with a reduction in force and joint compression, which will provide more oxygen consumption and energy expenditure for obese people (42). Unfortunately, there is a dearth of empirical study on the efficacy of the short-term aquatic HIIT training in improving the condition of overweight and obesity among adolescents (43). Furthermore, no comparative analysis has been conducted between aquatic HIIT and land HIIT on the biological and physiological variables. Therefore, the purpose of this study was to examine the effects of aquatic and land HIIT interventions on selected bio- and physiological variables in among obese adolescents. We hypothesize that: 1) Both Aquatic and Land HIIT interventions can improve body composition, physical function, blood pressure and lipid metabolism indexes among obese adolescents; 2) The aquatic HIIT would be superior to the Land HIIT due to aforementioned advantages of Aquatic HIIT for obese adolescents.

## 2 Materials and methods

### 2.1 Study design

**Figure 1:**
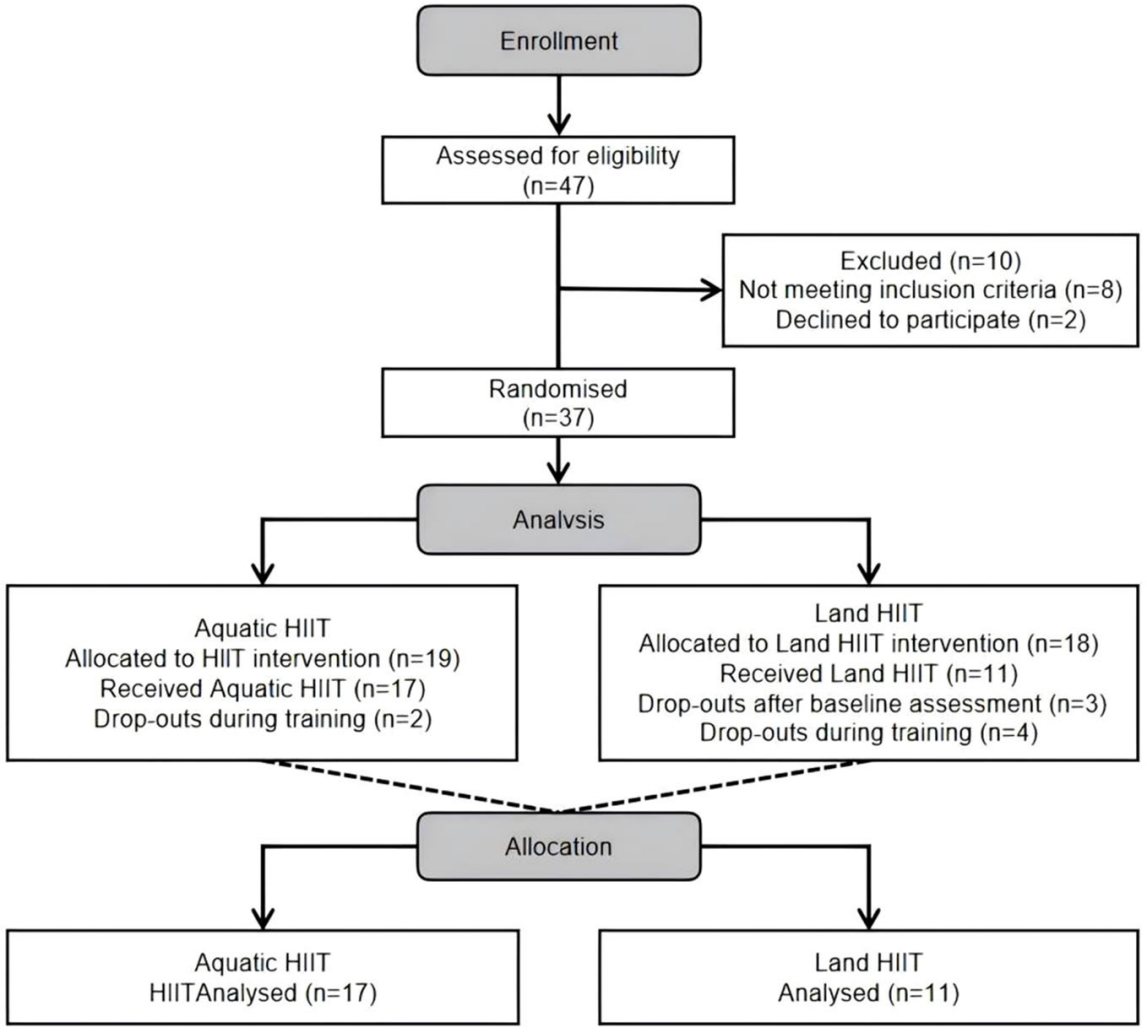
Flow-chart of the randomized trial.

The current study utilizes a randomized experimental design. Participants who met the inclusion criteria were randomly assigned to either the Aquatic HIIT group or the Land HIIT group in **Figure 1**. The exercise intervention was administrated for 4 weeks at a same location. The outcome measures including participants’ general demographic data, body composition, physical function, blood pressure and lipid metabolism were collected before and at the end of 4 week intervention. We developed the corresponding programs for the participants in different intervention groups, and a 60-minute aquatic HIIT session on every Monday, Wednesday, and Friday and a 60 minute land HIIT session on every Tuesday, Thursday, and Saturday, were administrated, respectively. These interventions were carried out at 5 pm on each scheduled day. This study was approved by the Ethics Board of Wuhan Sports University. The approval number is 20230521. This study meets the ethical standards for the journal (44).

### 2.2 Participants

Participants for this study were recruited in May 2021 at a youth weight loss camp in Wuhan, China. We used convenient sampling method and the participants were randomly assigned to the intervention group and control group. Inclusion criteria of the intervention (Aquatic HIIT) group and the control (Land HIIT) group were: 1) age between 10 and 18 years old, 2) body mass index (BMI) within the range of 24–35 kg/m^2^, 3) ability to participate in the whole intervention, and 4) no use of hypoglycemic, lipid-lowering or related drugs. Exclusion criteria included: 1) participation in a regular and structured physical activity plan within three months prior to the experiment, 2) recent hospitalization, 3) athletic disability, 4) symptomatic cardiopulmonary disease, 5) uncontrolled hypertension or metabolic syndrome, 6) severe kidney or liver disease, and 7) cognitive impairment or debilitating disease. Thirty-seven apparently healthy, obese adolescents were enrolled in this study. Twenty-eight participants (male, N = 15 and female, N = 13) eventually completed the study (Aquatic HIIT (n = 17); Land HIIT (n = 11) in **Table 1**. Prior to the commencement of the study, we explained to the participants and their parents about the study purpose and procedure in plain language prior to providing them the informed consent. The participants were informed of the experimental risks and the guardian signed an informed consent form for the participant prior to the study.

**Table 1:** Comparison of participant characteristics between Aquatic HIIT and Land HIIT before running program.

	Aquatic HIIT(n=17)	Land HIIT(n=11)	*p value*
Age (years)	12.76 ± 1.52	13.64 ± 1.69	0.168
Mass (kg)	82.65 ± 11.61	82.22 ± 12.26	0.925
Height (cm)	161.98 ± 6.83	164.61 ± 7.90	0.358
BMI(kg/m2)	31.44 ± 3.42	30.22 ± 2.93	0.341
Body fat (%)	33.90 ± 4.04	32.10 ± 3.33	0.230

Data are presented as mean ± SD.

### 2.3 Measurements

#### 2.3.1 Anthropometry and body composition

Height was measured in centimeters with the participant no shoes on, heels together, and standing back parallel to the height meter (RGZ-120-RT, Shanghai, China). Body mass was measured to an accuracy of 0.05 kg with the participant barefooted and wearing underwear or gym clothes standing on a weighing scale. Then, body mass index (BMI) is derived. All anthropometry measures were carried out according to international standards proposed by Lohman, Roche and Martorell (45). The body fat rate, lean body mass, body water content, waist circumference, hip circumference, and waist-to-hip ratio were measured using a multi-frequency bioelectrical impedance analyzer (Octapolar, InBody 520 model, South Korea).

#### 2.3.2 Physical function and blood pressure

The pulmonary function test which was performed with spirometry (Sensormedia Vmax Series 22, SEK) was used to determine the forced expiratory volume in one second (FEV1), forced expiratory capacity (FVC), FEV1/FVC and peak expiratory flow (PEF) (46). Blood pressure measurements were taken using an automated blood pressure meter (Omron BP652, Omron Healthcare Inc, Vernon Hills, IL, USA) with an appropriately sized cuff on the left arm. Participants were measured three times after a 5-minute rest in a sitting position with their legs uncrossed, and the average of the three measurements was recorded to measure resting systolic and diastolic blood pressures (SBP and DBP) (47).

#### 2.3.3 Lipid metabolism index

Fasting venous blood was collected in the morning before and after intervention (more than 48 hours from the last training). TC (TC), TG (TG), high-density lipoprotein cholesterol (HDL-C) and LDL-C (LDL-C) were measured on the automatic biochemical analyzer.

### 2.4 Intervention program

**Figure 2:**
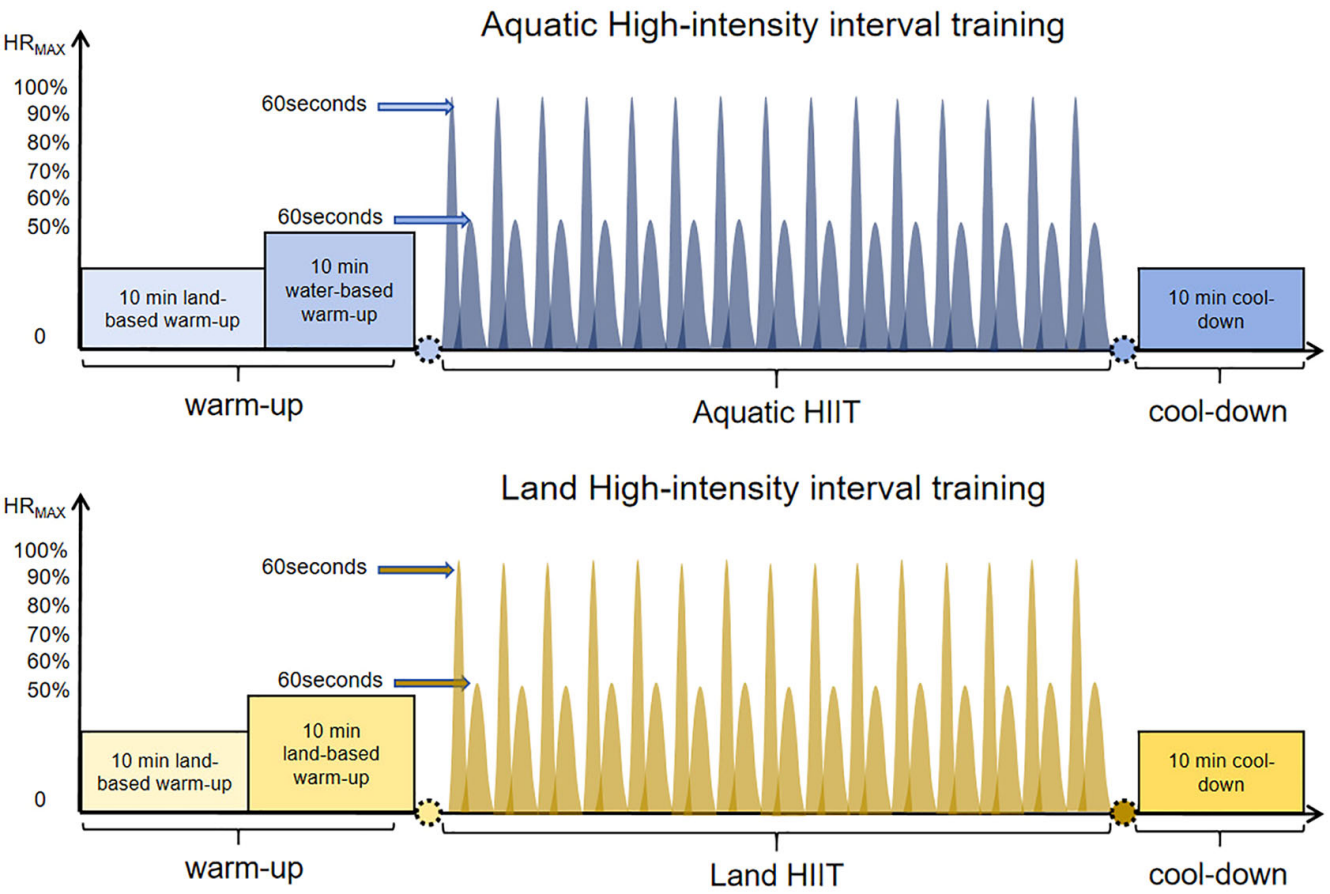
Aquatic and Land HIIT Intervention protocol.

The Participants received high intensity interval training during each training for 4 weeks, the flow of the aquatic-calisthenic high intensity interval training intervention is illustrated in **Figure 2** (48, 49). The water level was fixed at xiphoid level, with the temperature at 29~31°C. Participants wore a heart rate monitor (Polar, Büttelborn, Germany) and the heart rate was recorded during exercise. The entire intervention was overseen by researchers who actively encouraged the participants to ensure attainment of the target heart rate zone. The warm-up part consisted of 10 minutes of land dynamic stretching and 10 minutes of aquatic exercises with the warm-up heart rate controlled within (50–55% HRmax). Each (Aquatic HIIT or Land HIIT) group completed five movements (including Aquatic/Land Rocking Horse, Aquatic/Land Squat Jumps, Aquatic/Land Jumping Jacks, Aquatic/Land Cross Country Ski, and Aquatic/Land High Knees Run), respectively (50). When the exercise was performed for 1 minute and the heart rate was 80%—90% Max heart rate, the recovery period was set for 1 minute. During the interval between sets of exercise, the active recovery was adopted, such as jogging and walking slowly in the water. This part took a total of 30 minutes (37, 51). Finally, 10 minutes of static stretch relaxation on land was implemented (52). A 15-point Rating of Perceived Exertion (RPE) scale (48) was explained to the participants, and they were encouraged to exercise at an RPE of 17-18 for the aquatic high intensity intervals and at a rating of 11-12 for the active recovery intervals.

### 2.5 Statistical analysis

All data were expressed as mean standard deviation. Equality of variances was assessed using Levene’s test and normality was assessed using Shapiro-Wilk statistics. Nonparametric Kruskal-Wallis test was used when data was not normally distributed. All data passed the normality and homogeneity tests. The Mixed Model ANOVA was employed to examine the differences between and within the two groups using pre-test and post-test scores and the independent t-test was used to determine the improvements (post-test – pre-test) of all the variables between the two groups. The level of significant difference between the comparisons was set up at *p*<0.05. Data analysis was performed by means of the SPSS Statistical Software (v20.0; SPSS Inc., Chicago, IL, USA).

## 3 Results

### 3.1 Anthropometry and body composition

The statistical results of anthropometry and body composition of aquatic HIIT and land HIIT are presented in **Table 2**. There were no significant differences among these variables between the two groups before the interventions (*p*>0.05). After four weeks of exercise interventions, the mass, BMI, body fat rate, waist circumference, hip circumference and body water content were significantly reduced (*p*<0.05), while the lean body mass were significantly increased (*p <*0.05) in both groups. Furthermore, it can be seen that the Aquatic HIIT group had significant improvements in Lean body mass, waist circumference, and waist-to-hip ratio than Land HIIT group did in **Table 3**.

**Table 2:** The anthrpometry and body composition of participants pre- vs. post-training programs (mean ± SD).

	Aquatic HIIT(n=17)		*p*-value	Land HIIT(n=11)		*p*-value
	Pre-training	Post-training		Pre-training	Post-training	
Height (cm)	161.98 ± 6.83	162.13 ± 6.95	0.206	164.61 ± 7.90	164.84 ± 7.81	0.134
Mass (kg)	82.65 ± 11.61	81.41 ± 10.97^*^	0.006	82.22 ± 12.26	80.31 ± 11.96^*^	0.020
BMI (kg/m2)	31.44 ± 3.42	30.94 ± 3.34^*^	0.004	30.22 ± 2.93	29.45 ± 2.92^*^	0.008
Fat (%)	33.90 ± 4.04	29.62 ± 4.98^*^	<0.001	32.10 ± 3.33	28.55 ± 3.93^*^	0.001
Lean body mass (kg)	54.33 ± 5.91	54.96 ± 5.66^*^	0.038	55.66 ± 7.73	57.57 ± 7.59^*^	0.001
Body water content (%)	39.12 ± 4.25	37.69 ± 4.12^*^	<0.001	40.07 ± 5.57	38.62 ± 5.15^*^	0.001
Waist circumference (cm)	87.14 ± 4.93	83.88 ± 4.36^*#^	<0.001	90.22 ± 4.15	88.27 ± 4.99^*#^	0.004
Hip circumference (cm)	100.16 ± 6.31	99.04 ± 6.57^*^	0.028	100.66 ± 6.04	99.03 ± 5.72^*^	0.001
Waist hip ratio (%)	0.87 ± 0.06	0.85 ± 0.06^*#^	<0.001	0.90 ± 0.02	0.89 ± 0.02^#^	0.395

Data are presented as mean ± SD.

*p < 0.05, post-test versus pretest within groups.

#*p* < 0.05, Aquatic HIIT group (post-test) vs. Land HIIT group (post-test).

**Table 3:** The difference of anthropometry and body composition between before and after intervention of Aquatic HIIT and Land HIIT groups (mean ± SD).

	Subtracting pre-training values from post-training values.		
	Aquatic HIIT	Land HIIT	*p*-value
Height (cm)	0.15 ± 0.46	0.23 ± 0.46	0.657
Mass (kg)	-1.24 ± 1.60	-1.91 ± 2.30	0.372
BMI (kg/m2)	-0.50 ± 0.61	-0.77 ± 0.78	0.312
Fat (%)	-4.28 ± 1.45	-3.55 ± 1.69	0.234
Lean body mass (kg)	0.63 ± 1.15^**^	1.91 ± 0.69^**^	0.003
Body water content (%)	-1.42 ± 0.83	-1.45 ± 0.88	0.925
Waist circumference (cm)	-3.25 ± 1.05^*^	-1.95 ± 1.77^*^	0.020
Hip circumference (cm)	-1.12 ± 1.92	-1.64 ± 0.97	0.421
Waist hip ratio (%)	0.26 ± 0.43^**^	0.89 ± 0.02^**^	0.001

Data are presented as mean ± SD; *p<0.05, and **p<0.01.

### 3.2 Physical function and blood pressure

The changes in physical function and blood pressure before and after interventions in both groups are presented in **Table 4**. The Aquatic HIIT group exhibited significant decreases in Rest HR (*p*<0.05), systolic blood pressure (SBP) (*p*<0.05), diastolic blood pressure (DBP) (*p*<0.01), and significant increases in vital capacity and total energy consumption (*p*<0.05). The Land HIIT group showed significant decreases in SBP (*p*<0.05) and DBP (*p*<0.01), significant increases in vital capacity (*p*<0.01) and total energy expenditure (*p*<0.05), but no significant changes in Rest HR (*p*=0.364). In addition, it can be seen that the Aquatic HIIT group had significant improvements in vital capacity, and total energy consumption than Land HIIT group did in **Table 5**.

**Table 4:** The physical function and blood pressure of participants pre- vs. post-training programs (mean ± SD).

Parameters	Aquatic HIIT(n=17)		*p*-value	Land HIIT(n=11)		*p*-value
	pre-training	post-training		pre-training	post-training	
Vital capacity (ml)	2286.88 ± 498.02	3013.00 ± 545.03^*#^	<0.001	2342.55 ± 505.03	2578.18 ± 483.37^*#^	0.005
Rest HR (Bpm)	70.76 ± 7.15	67.06 ± 5.83^*#^	0.025	74.18 ± 4.90	73.09 ± 5.58^#^	0.364
SBP (mmHg)	110.82 ± 10.55	108.29 ± 8.14^*^	0.028	112.36 ± 11.05	108.45 ± 11.06^*^	0.001
DBP (mmHg)	69.94 ± 7.34	66.29 ± 6.54^*^	0.006	66.55 ± 6.15	61.73 ± 3.77^*^	0.011
Total energy consumption (kcal)	2073.71 ± 178.94	2316.24 ± 135.89^*#^	<0.001	2083.18 ± 162.30	2190.91 ± 155.19^*#^	0.012

Data are presented as mean ± SD; SBP, systolic blood pressure; DBP, diastolic blood pressure; HR, heart rate.

*p < 0.05, post-test versus pretest within groups.

#*p* < 0.05, Aquatic HIIT group (post-test) vs. Land HIIT group (post-test).

**Table 5:** The difference of physical function and blood pressure between before and after intervention of Aquatic HIIT and Land HIIT groups (mean ± SD).

Parameters	subtracting pre-training values from post-training values		
	Aquatic HIIT	Land HIIT	*p*-value
Vital capacity (ml)	726.12 ± 193.42^**^	235.64 ± 215.12^**^	0.001
Rest HR (Bpm)	-3.71 ± 6.20	-1.09 ± 3.81	0.223
SBP (mmHg)	-2.53 ± 4.30	-3.91 ± 2.55	0.348
DBP (mmHg)	-3.65 ± 4.69	-4.82 ± 5.15	0.540
Total energy consumption (kcal)	242.53 ± 80.92^**^	107.73 ± 117.34^**^	0.001

Data are presented as mean ± SD; SBP, systolic blood pressure; DBP; diastolic blood pressure; HR, heart rate; **p<0.01.

### 3.3 Lipid metabolism index

The results of the TC, TG, HDL-C and LDL-C are presented in **Table 6**. The LDL-C in Aquatic HIIT and Land HIIT groups were significantly decreased (*p*<0.05), However, there was no significant change in other Lipid metabolism indexes, and no statistically significant differences were found between the groups across all indicators. Furthermore, it can be seen that there is not significant changes between Aquatic HIIT group and Land HIIT group in terms of the improvements in TC, TG, HDL-C and LDL-C in **Table 7**.

**Table 6:** The lipid metabolism index of participants pre- vs. post-training programs (mean ± SD).

	Aquatic HIIT(n=17)		*p*-value	Land HIIT(n=11)		*p*-value
	Pre-training	Post-training		Pre-training	Post-training	
Total cholesterol (mmol/L)	4.45 ± 0.72	4.42 ± 0.73	0.087	4.69 ± 0.70	4.64 ± 0.74	0.052
Triglyceride (mmol/L)	0.95 ± 0.21	0.94 ± 0.25	0.304	0.99 ± 0.12	0.98 ± 0.15	0.706
HDL-cholesterol (mmol/L)	1.33 ± 0.17	1.34 ± 0.20	0.417	1.34 ± 0.33	1.39 ± 0.36	0.053
LDL-cholesterol (mmol/L)	2.78 ± 0.62	2.69 ± 0.65^*^	0.002	2.89 ± 0.74	2.80 ± 0.71^*^	0.001

Data are presented as mean ± SD; HDL, high-density lipoprotein; LDL, low-density lipoprotein.

*p < 0.05, post-test versus pretest within groups.

#*p* < 0.05, Aquatic HIIT group (post-test) vs. Land HIIT group (post-test).

**Table 7:** The difference of lipid metabolism index between before and after intervention of Aquatic HIIT and Land HIIT groups (mean ± SD).

	Subtracting pre-training values from post-training values		
	Aquatic HIIT	Land HIIT	*p*-value
Total cholesterol (mmol/L)	-0.03 ± 0.07	-0.05 ± 0.07	0.580
Triglyceride (mmol/L)	-0.02 ± 0.07	-0.01 ± 0.05	0.610
HDL-cholesterol (mmol/L)	0.01 ± 0.06	0.04 ± 0.06	0.208
LDL-cholesterol (mmol/L)	-0.09 ± 0.09	-0.10 ± 0.06	0.744

Data are presented as mean ± SD; HDL, high-density lipoprotein; LDL, low-density lipoprotein.

## 4 Discussion

This study aimed to examine the effects of Aquatic HIIT vs Land HIIT on body composition, physical function, blood pressure and lipid metabolism index among obese adolescents. The results of the present study showed that the hypotheses were valid, and the two exercise interventions improved body composition, Physical function, blood pressure and lipid metabolism indexes in overweight and obese adolescents to varying degrees. The results from the present study are consistent with the research results of Zhu, Ying, Delgado-Floody et al. (11, 17). Moreover, the Aquatic HIIT group had significantly better improvement effects on lean body mass, waist circumference, waist-to-hip ratio, vital capacity, and total energy consumption compared with the Land HIIT did. Based on our research design, this study was the first one to explore the Aquatic HIIT vs Land HIIT in a short-term and high-volume, and evaluate the results immediately after the interventions. The results of the present study demonstrated the similar effects of the short-term high-volume of the Aquatic HIIT and Land HIIT on obese adolescents.

It is worth noting that different exercise intervention schemes and procedures may affect the experimental results, however, by using the same cycle, exercise intensity, duration, and frequency, aquatic exercise may achieve similar results to land-based exercise in improving body composition (39). Therefore, we may adapt an exercise intervention scheme of HIIT as an aquatic exercise, to improve aerobic capacity and body composition of adolescences. Consequently, the purpose of this study is to explore the possible implementation of converting the Land HIIT to the aquatic environment with the high-intensity interval training of aquatic-calisthenics. That is why we kept the exercise intensity, duration and frequency of the intervention programs in each (Aquatic or Land HIIT) group identical. The results showed there were no significant differences in body composition, physical function, blood pressure, and lipid metabolism index between two groups after intervention, which might be due to the high similarity of intervention regimens between the two groups. Although recent evidence linking participation in Land HIIT to a greater risk of injury (53), performing HIIT in an aquatic environment may reduce joint pressure, make the Aquatic HIIT a safer form of exercise, and motivate the exercise participation and enthusiasm of the overweight and obese people (34–36). Furthermore, the aquatic-calisthenic high intensity interval training may be employed to improve the cardiopulmonary function and body composition of Sedentary Young Adults (50).

### 4.1 Physical variables

Overweight and obesity are mainly characterized by adverse changes in body composition, which will not only cause metabolic disorders, but also negatively affect the cardiovascular function (54, 55). The results of the present study showed that Land HIIT group could reduce body fat and improve body composition, such results are supported by the previous studies (12, 13, 15). Some exercise interventions such as aerobic exercises for the treatment of obesity in adolescents have shown improvements in body composition, and these findings are consistent with the results of the present study, which demonstrated the reductions in body fat, BMI, and body fat percentage (56, 57). The results from the previous studies have demonstrated positive effects in body composition, including reduced body fat and improved muscle mass, as a result of water-based exercise (58). Changes in body composition during water-based exercise may be attributed to the extra caloric expenditure related to the water resistance. Oxygen consumption (VO2) values during water training are higher than those during land-based training at equivalent intensities, resulting in greater caloric expenditure. Therefore, if water exercise induces higher VO2 values, it should lead to increased caloric expenditure over time and consequent reduction of body fat (59). However, it is noteworthy that the intervention cycles of the previous studies were more than 12 weeks, whereas, the results of the present study showed that a short-term (4-week) high-volume Aquatic HIIT could also effectively improve the body composition of obese adolescents, and had similar effects to the Land HIIT in improving the body mass, BMI, body fat rate, lean body mass, body water content, and hip circumference of the obesity adolescents. Moreover, the Aquatic HIIT group demonstrated significantly better effects than the Land HIIT group on improving the lean body mass, waist circumference, and waist hip ratio of the obesity adolescents.

### 4.2 Physiological variables

Previous studies have shown that the effect of aquatic environment on the improvement of vital capacity is more obvious than that of land environment (60, 61), at the same time, the results of the present study showed that the Aquatic HIIT improved vital capacity significantly better than Land HIIT did. This phenomenon may be attributed to the hydrostatic pressure and viscous force exerted by water, which generate enhanced abdominal compression through increased pressure and resistance. To move in the water the respiratory muscles need to overcome the greater resistance and pressure during the exercise with comparison to the land exercise.

The results of the present study showed that the Aquatic HIIT could reduce resting heart rate of the obese adolescents, but the Land HIIT did not achieve this effect. This is similar to the findings of Igarashi (62), which suggest that aquatic exercise can effectively enhance parasympathetic nerve activity, reduce the tension in the sympathetic nervous system during rest, and lower the resting heart rate by an average of 5.2 beats per minute in **Table 4** in the present study.

In recent years, many researchers have proposed that high intensity interval training is more effective than medium or low-intensity training in lowering blood pressure, and it is a safe and effective way to interfere with hypertension (63). The present study verified the results of the previous research and found that the Aquatic HIIT can also effectively reduce the diastolic blood pressure of the obese adolescents. There are several possible antihypertensive mechanisms of exercise in aquatic. First, hydrostatic pressure can affect the stimulation of baroreceptors, and hydrostatic pressure promotes venous return and stimulates baroreceptors, triggering the increases in cardiac fullness and stroke volume, which reflexively lowers blood pressure (64). Secondly, water temperature can make a difference, and the water temperature in the present study ranged 29~31°C, and the previous studies have shown that the water temperature around 30~32°C may cause arteriolar dilation, resulting in a reduction in peripheral vascular resistance (65, 66), thus achieving the antihypertensive effect. Additionally, aquatic environment is associated with an increase in the concentration of nitric oxide in the blood. Studies have shown that aquatic exercise can increase the concentration of nitric oxide and improve vascular endothelial function, so that the aquatic exercise can promote vascular relaxation to achieve a reduction in blood pressure (67, 68). Decrease of diastolic blood pressure in the obese adolescents is also related to the sympathetic nervous system; aquatic exercise may decrease activity within the human body’s sympathetic nervous system, and produce an antihypertensive effect (69).

### 4.3 Biological variables

Lipid metabolism indexes are one type of the biomarkers of metabolic disorders in obese people. For example, elevated plasma LDL-C and TG concentrations are positively correlated with the incidence of coronary heart disease (70), and low HDL-C concentrations are a risk factor for coronary atherosclerosis (55, 71, 72). A few studies have shown that the HIIT can significantly improve the lipid metabolism indexes of obese people, such as reducing TC, TG, LDL-C, blood glucose, insulin, and increasing HDL-C (73, 74). However, in the present study, it was found that both the Aquatic HIIT group and the Land HIIT group could only reduce LDL-C concentrations without significant effects on other lipid metabolism indexes. Consistent with the result of the present study, Ouerghi et al. (75) found that 8 weeks of HIIT (load intensity 100%-110% Maximal Aerobic Speed, duration 30s) significantly reduced LDL-C in overweight and obese youth (BMI 30.8 ± 4.6 kg/m2), so did Racil et al. (76). Furthermore, significant decreases in TC and TG were also observed among the participants from both studies (75, 76). However, some researchers have presented different results, such as Sawyer et al. (77), found that after 8 weeks of HIIT (load intensity of 90%–95% max heart rate and load time of 60s), TC, TG, LDL-C and HDL-C of obese adults showed no significant changes. Khammassi (78) and Smith et al. (79) have studied overweight and obese adults and reached similar conclusions. A relevant meta-analysis (80–82) has shown that the HIIT can effectively improve the insulin sensitivity of overweight and obese young people, healthy young woman and adult men with metabolic syndrome. However, the improvements in Lipid metabolism indexes varied depending on the different HIIT regimens. For example, a short-term (≤4 weeks) sprint interval training has no significant improvement on Lipid metabolism indexes, while a long-term (≥12 weeks) HIIT intervention may have a positive effect on Lipid metabolism indexes (83). These differences may be related to factors such as the diversity of training programs (intensity of load, duration, form of exercise, etc.), measurement techniques, gender (male or female), age difference (children, adolescents, adults or the elderly), or the degree of obesity (moderate, severe or morbid) (8, 14). However, the specific mechanism of the Aquatic HIIT or Land HIIT for improving glucose and lipid metabolism index is not well elaborated and interpreted. Some studies have proposed the possible mechanism of HIIT-induced adaptive changes in the body, including its efficacy in promoting the catabolism of fat (84, 85), increasing excessive oxygen consumption after intensive exercise (86, 87) and inhibiting appetite (88).

### 4.4 Limitations

The limitations of the present study are: 1) the sample size of Aquatic HIIT or Land HIIT groups was small, and the attrition rate is relative high, especially for the Land HIIT group so that we should increase the sample size and manage to reduce the attrition rate in the future study; 2) the influence of gender on morphology and physical has not been taken into consideration so that with sufficient number of participants, the effect of Aquatic and Land HIITs effect males and females during adolescence should be examined; 3) although we supervised and closely monitored for adherence, no other exercise or food intake data were collected in two groups to confirm that they had not changed their behavior during the 4 weeks; 4) 4 week intervention period might be too short and the long term effects of Aquatic HIIT and Land HIIT should be examined; and 5) the interrelationship between exercise and changes in body composition makes it difficult to pinpoint specific contributors (whether directly or indirectly) on the physiological changes observed in the current study.

## 5 Conclusion

The results of the present study demonstrated that a short-term (4 weeks) Aquatic HIIT may be effective in improving the body composition, physical function, blood pressure and LDL-C of overweight and obese adolescents, and may have similar effects as the Land HIIT did. Furthermore, the Aquatic HIIT is more significantly effective than the Land HIIT in improving waist circumference, waist hip ratio, and resting heart rate. It should be noted that in the present study, the aquatic-calisthenic high intensity interval training, as a non-weight-bearing aerobics exercise, utilized the buoyancy and resistance of the aquatic environment to reduce body weight load, without the need of additional auxiliary devices, and HIIT may be an optional exercise for overweight and obese adolescents. It is recommended that further research focus on aquatic high intensity interval training with different cycles, exercise intensity, duration and frequency on different overweigh and obese populations such as gender and age, to optimize the exercise intervention scheme for improving the health problems of overweight and obese adolescents and adults.

## Data Availability

The original contributions presented in the study are included in the article/**Supplementary Material**. Further inquiries can be directed to the corresponding author.
